# Clinical Outcomes of Transradial Versus Transfemoral Approach in Rotational Atherectomy: Results from the Rotational Atherectomy in Calcified Lesions in Korea (ROCK) Registry

**DOI:** 10.3390/jcm14093066

**Published:** 2025-04-29

**Authors:** Kyunyeon Kim, Jin Jung, Sung-Ho Her, Kyusup Lee, Ji-Hoon Jung, Ki-Dong Yoo, Keon-Woong Moon, Donggyu Moon, Su-Nam Lee, Won-Young Jang, Ik-Jun Choi, Jae-Hwan Lee, Jang-Hoon Lee, Sang-Rok Lee, Seung-Whan Lee, Kyeong-Ho Yun, Hyun-Jong Lee

**Affiliations:** 1 Department of Cardiology, St. Vincent’s Hospital, The Catholic University of Korea, Seoul 16247, Republic of Korea; gemini729@naver.com (K.K.); hhhsungho@naver.com (S.-H.H.); cardioyoo@gmail.com (K.-D.Y.); cardiomoon@gmail.com (K.-W.M.); donggoo@catholic.ac.kr (D.M.); yellow-night@hanmail.net (S.-N.L.); raph83@naver.com (W.-Y.J.); 2 Department of Cardiology, Daejeon St. Mary’s Hospital, College of Medicine, The Catholic University of Korea, Seoul 34943, Republic of Korea; ajobi7121@gmail.com; 3 Korea Institute of Toxicology, Daejeon 34114, Republic of Korea; jihoon.jung@kitox.re.kr; 4 Department of Cardiology, Incheon St. Mary’s Hospital, College of Medicine, The Catholic University of Korea, Incheon 21431, Republic of Korea; mrfasthand@catholic.ac.kr; 5 Department of Cardiology in Internal Medicine, Chungnam National University School of Medicine, Daejeon 35015, Republic of Korea; myheart@cnu.ac.kr; 6 Department of Internal Medicine, Kyungpook National University Hospital, Daegu 41944, Republic of Korea; ljhmh75@knu.ac.kr; 7 Department of Cardiology, Chonbuk National University Hospital, Jeonju 54907, Republic of Korea; medorche@jbnu.ac.kr; 8 Department of Cardiology, Asan Medical Center, University of Ulsan College of Medicine, Seoul 05505, Republic of Korea; seungwlee@amc.seoul.kr; 9 Department of Cardiovascular Medicine, Regional Cardiocerebrovascular Center, Wonkwang University Hospital, Iksan 54538, Republic of Korea; dryunkh@gmail.com; 10 Department of Internal Medicine, Sejong General Hospital, Bucheon 14754, Republic of Korea; untouchables00@hanmail.net

**Keywords:** atherectomy, mortality, hemorrhage, coronary vessels

## Abstract

**Background and Objectives:** Rotational atherectomy (RA) is a crucial method for percutaneous coronary intervention (PCI) of heavily calcified coronary lesions. The aim of this study was to compare the clinical outcomes in patients undergoing RA via the radial versus femoral approach. **Methods:** The Rotational Atherectomy in Calcified Lesions in Korea (ROCK) registry included consecutive patients with severely calcified coronary artery disease who received RA during PCI at nine tertiary centers in Korea. A total of 540 patients who underwent PCI with RA were enrolled between October 2019 and January 2010. We retrospectively investigated the clinical outcomes between the transradial and transfemoral approaches. The primary endpoint was major adverse cardiac and cerebrovascular events (MACCE) within 36 months of follow-up. **Results:** Of the 540 patients, 248 patients (45.9%) were in the transradial group, and 292 patients (54.1%) were in the transfemoral group. There were no significant differences in MACCE (11.3% vs. 17.8%, adjusted hazard ratio [HR]: 1.520; 95% confidence interval: 0.889–2.600; *p* = 0.126) and procedural success (97.6% vs. 95.2%, *p* = 0.145). The occurrence of in-hospital bleeding was numerically higher in the transfemoral group, but the difference was not statistically significant (8 [3.2%] vs. 19 [6.5%], *p* = 0.081) **Conclusions:** In this study, the transradial approach did not show a significant difference in clinical outcomes but tended to have lower bleeding events compared to the transfemoral approach. RA via the transradial approach can be a useful vascular access option compared to the transfemoral approach.

## 1. Introduction

Heavily calcified coronary lesions continue to present substantial difficulties during percutaneous coronary interventions (PCI) due to challenges in lesion crossing, inadequate stent expansion, and a higher risk of procedural complications such as dissection or perforation [1,2]. As life expectancy rises and the population ages, an increasing number of patients are being referred for PCI to manage these complex lesions [3]. Rotational atherectomy (RA), first introduced in 1989, remains an essential method for addressing such heavily calcified and resistant-to-dilation lesions [4].

Both transradial (TR) and transfemoral (TF) approaches are feasible for PCI using rotational atherectomy. In conventional PCI, previous studies have consistently demonstrated better outcomes with the TR approach compared to the TF approach [5,6]. Additionally, the 2023 ESC guidelines recommend radial access as the preferred approach [7]. However, traditionally, the TF approach has been the preferred method for RA, primarily because large-caliber guide catheters are needed to accommodate the atherectomy burrs. The TF approach provides greater support, particularly when using larger burr sizes [8]. However, initially used for plaque debulking, RA has since transitioned into a technique for plaque modification, and the use of large-sized burrs has decreased compared to the past [9]. With advancements in device technology, most burr sizes are now compatible with the TR approach. The TR approach has become increasingly popular in coronary angiography and PCI due to its association with fewer vascular and bleeding complications, as well as enhanced patient comfort and quicker mobilization [5,6].

Most existing studies focus on the general benefits of the TR approach in PCI, such as lower mortality rates and reduced major vascular complications, but few have specifically examined its use in RA [10,11,12]. To the best of our knowledge, there is no data on Asians. Considering that East Asians have a higher bleeding risk compared to Western populations, it is even more important to compare the outcomes and safety of the TR and TF approaches in Asian patients [13]. This study aims to compare the clinical outcomes of patients undergoing RA through the TR versus the TF approach.

## 2. Methods

### 2.1. Study Design and Population

From January 2010 to October 2019, this study enrolled 540 patients with heavily calcified coronary artery disease (CAD) who underwent PCI using RA at nine tertiary centers in Korea. The data were derived from the Rotational Atherectomy in Calcified Lesions in Korea (ROCK) registry, approved by the institutional review board of each participating hospital. Patients with severely calcified coronary lesions and significant stenosis (defined as ≥70% of the vessel diameter, or ≥50% for the left main coronary artery) were retrospectively identified through institutional databases. Following angiographic assessment, two lesions were excluded from the registry because the RA procedure could not be carried out. In one case, coronary perforation accompanied by cardiac tamponade occurred prior to the initiation of RA. In the other, advancement of the guidewire across the target lesion was unsuccessful, making RA unfeasible. The lesions were categorized into two groups: transradial approach (*n* = 248 patients) and transfemoral approach (*n* = 292 patients). Data collection at each center followed a standardized case report form, capturing demographic and clinical characteristics, procedural details, and follow-up information. Follow-up data covering up to 36 months were obtained from medical records and physician or patient interviews conducted at the time of registry enrollment. The study was conducted in accordance with the Declaration of Helsinki and was approved by the Institutional Review Board (IRB) of Daejeon St. Mary’s Hospital (Approval Code: DC19REDI0066, approved on 30 July 2019).

### 2.2. RA Procedure

All RA procedures were performed using the Rotablator™ RA system (Boston Scientific, Marlborough, MA, USA). The procedural techniques and treatment approaches followed those outlined in previous reports [14]. The choice of burr size and other procedural decisions were made by the treating physician, considering the complexity of the anatomy, the patient’s overall clinical condition, and relevant clinical risk factors. During the follow-up period, patient care, including the administration of periprocedural anticoagulation and antiplatelet therapy, adhered to established guidelines and standard medical practices.

### 2.3. Clinical Outcomes and Definition

The primary outcome was major adverse cardiac and cerebrovascular events (MACCE), defined as a composite of cardiac death, target vessel spontaneous myocardial infarction (TVMI), and target vessel revascularization (TVR). Cardiac death was defined as any mortality resulting from cardiac-related causes, including myocardial infarction, heart failure, arrhythmia, or other cardiovascular complications. Secondary endpoints included all-cause death, cardiac death, any MI, TVMI, TVR, stent thrombosis (ST), cerebrovascular accident (CVA), and total bleeding. Total bleeding was defined as the sum of all bleeding events classified according to the Bleeding Academic Research Consortium (BARC) criteria, including types 1 through 5. Additionally, technical and procedural success, in-hospital events, and periprocedural complications were evaluated. The definitions of the outcomes were consistent with those in the previously cited published report [14].

### 2.4. Statistical Analysis

Continuous variables were analyzed using the t-test and reported as medians with interquartile ranges or means with standard deviations. Categorical variables were compared using the chi-square test or Fisher’s exact test and presented as frequencies and percentages. Cox proportional hazard models were applied to assess the impact of vascular access on clinical outcomes. Multivariate Cox regression analyses were performed using variables with a *p*-value < 0.1 in the univariate analyses. Hazard ratios (HR) and 95% confidence intervals (CI) were calculated. Clinical outcomes were estimated using the Kaplan–Meier method and compared with the log-rank test. A *p*-value < 0.05 was considered statistically significant. The multivariate Cox regression models were adjusted for the following covariates: age, sex, chronic kidney disease (CKD), dialysis, previous coronary artery bypass graft (CABG), peripheral vascular disease (PVD), history of heart failure, left ventricular ejection fraction (EF), hemoglobin level, total cholesterol, and low-density lipoprotein (LDL) cholesterol. All statistical analyses were carried out using Statistical Analysis Software (SAS, version 9.2, SAS Institute, Cary, NC, USA).

## 3. Results

### 3.1. Baseline Characteristics

**Figure 1:**
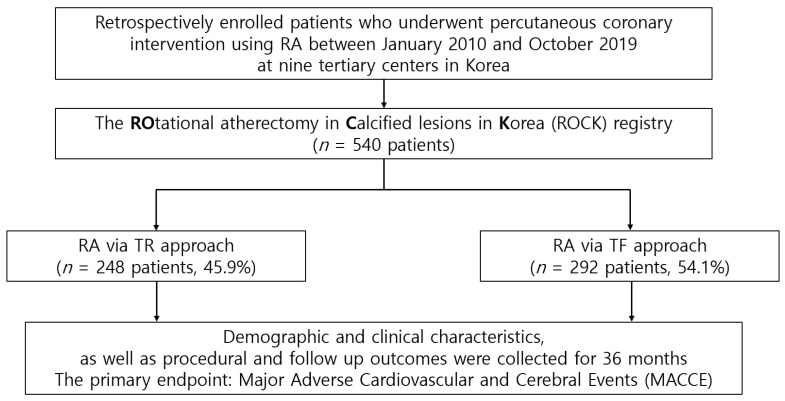
Study population flow chart. RA = rotational atherectomy; TF = transfemoral; TR = transradial.

Patients were divided into two groups according to the vascular approach for RA. Among a total of 540 patients, 248 patients (45.9%) were in the TR approach group, and 292 patients (54.1%) were in the TF approach group. The study population flow chart is presented in Figure 1. Table 1 presents a comparison of baseline characteristics between the TR and TF approach groups. The mean age was 72.5 years for the TR group, compared to 70.5 years for the TF group, with a statistically significant difference (*p* = 0.024). There were no statistically significant differences between the two groups in terms of gender, body mass index (BMI), history of smoking, prevalence of diabetes mellitus (DM), hypertension (HTN), previous PCI, or MI. Comorbidities, including CKD, dialysis, previous CABG, PVD, and the history of heart failure, were more common in the TF approach group. The TF group had lower levels of hemoglobin. The EF of the left ventricle was significantly lower in the TF group (Table 2).

### 3.2. In-Hospital Events and Procedural Outcomes

Table 3 summarizes the in-hospital events and procedural outcomes between the TF approach group and the TR approach group. There were no significant differences in procedural success between the groups (97.6% vs. 95.2%, *p* = 0.145). For in-hospital events and procedural outcomes, there were no statistically significant differences except for the insertion of a temporary pacemaker during the procedure (*p* = 0.007). In-hospital bleeding had a higher tendency in the TF approach group (3.2% vs. 6.5%, *p* = 0.081).

### 3.3. Clinical Outcomes

**Figure 2:**
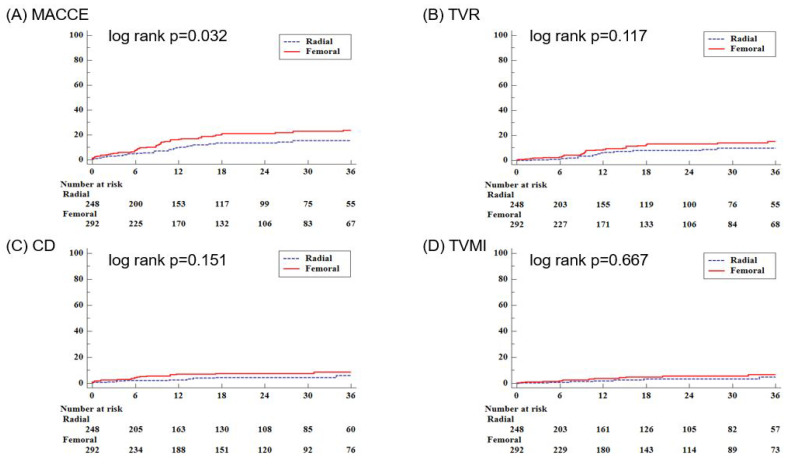
The Kaplan–Meier curves for clinical outcomes during the follow-up period, including the primary outcome of MACCE and its components. CD = cardiac death; MACCE = major adverse cardiac and cerebrovascular events; TVMI = target vessel spontaneous myocardial infarction; TVR = target vessel revascularization.

Figure 2 shows the Kaplan–Meier curve for clinical outcomes during follow-up. During the 36-month follow-up period, MACCE showed a statistically significant difference between the two groups with a *p*-value of 0.032. No significant differences were observed in CD, TVMI, or TVR. However, in the multivariate analysis of MACCE at 36 months, there were no significant differences between the TF approach group and the TR approach group (unadjusted HR: 1.643, 95% CI: 1.038–2.601, *p* = 0.034; adjusted HR: 1.520, 95% CI: 0.889–2.600, *p* = 0.126) (Table 4). The secondary endpoints also did not show significant differences (all-cause death: unadjusted HR: 1.124, 95% CI: 0.627–2.013, *p* = 0.695; adjusted HR: 1.230, 95% CI: 0.610–2.482, *p* = 0.562; any MI: unadjusted HR: 1.639, 95% CI: 0.654–4.108, *p* = 0.292; adjusted HR: 1.601, 95% CI: 0.536–4.786, *p* = 0.400; TLR: unadjusted HR: 1.706, 95% CI: 0.873–3.334, *p* = 0.118; adjusted HR: 1.245, 95% CI: 0.582–2.663, *p* = 0.572; ST: unadjusted HR: 2.163, 95% CI: 0.420–11.151, *p* = 0.356; adjusted HR: 2.224, 95% CI: 0.343–14.414, *p* = 0.402; CVA: unadjusted HR: 1.301, 95% CI: 0.367–4.610, *p* = 0.684; adjusted HR: 1.479, 95% CI: 0.230–9.512, *p* = 0.681; total bleeding: unadjusted HR: 1.280, 95% CI: 0.632–2.592, *p* = 0.493; adjusted HR: 1.214, 95% CI: 0.526–2.802, *p* = 0.649).

## 4. Discussion

The main findings of this study are as follows: (1) The TR approach demonstrated comparable outcomes to the TF approach in terms of MACCE. (2) In-hospital events and procedural outcomes showed no statistically significant differences between the two groups; however, in-hospital bleeding had a higher tendency in the TF approach group.

PCI performed via the TR approach has been shown to reduce vascular and bleeding complications while improving clinical outcomes in a wide range of patients with coronary artery disease compared to the TF approach [5,6]. Consequently, it has been recommended as the preferred access site in the most recent European guidelines on myocardial revascularization [7]. With the aging population, heavily calcified coronary lesions are becoming more common, resulting in a growing need for RA in these cases [4,15]. The TR approach is used less frequently in complex PCI procedures, such as those requiring RA, compared to the general PCI population, due to the need to accommodate larger guiding catheters and provide greater backup support [8,16]. However, the TF approach is associated with a higher risk of bleeding complications, a concern that is particularly pronounced in East Asian populations, where the risk of bleeding often outweighs the risk of ischemic events [13]. This elevated bleeding risk associated with the TF approach may be one of the factors contributing to hesitancy in adopting RA, despite its necessity for managing severely calcified lesions [17,18]. Confirming that the TR approach in RA does not result in significant differences in clinical outcomes would highlight its advantage in reducing bleeding risks. Furthermore, this finding suggests the possibility of the TR approach becoming a safe and efficient vascular access strategy in RA as well. In this context, distal radial access (DRA) is also gaining attention as a feasible and safe option for RA. Recent studies have demonstrated its applicability in complex procedures such as complex, high-risk indicated PCI, with potential advantages including reduced bleeding and enhanced patient comfort. Moreover, DRA may even facilitate radial artery occlusion recanalization, thus preserving long-term vascular access. While our registry did not specifically distinguish between conventional and distal radial access, this technique represents a promising advancement in access strategies for high-risk indicated PCI [19].

Our study demonstrated that performing RA via the TR approach resulted in clinical outcomes comparable to those of the TF approach, with a tendency for lower in-hospital bleeding. These findings support the broader use of TR with RA even in Asian populations. Similar results have also been reported in previous studies conducted in Western populations [20,21,22,23]. A meta-analysis demonstrated that the TR approach for RA of calcified native coronary lesions is associated with a lower incidence of access site bleeding, while achieving comparable procedural success, procedural time, all-cause mortality, and major adverse cardiac events compared to the TF approach [20]. Similarly, a study found that the TR approach was associated with a significantly lower risk of bleeding complications without compromising angiographic success or long-term efficacy compared to the TF approach [21]. Also, small single-center observational studies showed that the TR approach was associated with similar procedural success but lower risk for in-hospital bleeding and vascular complications compared to the TF approach [22,23]. Our findings are in line with those of previous studies.

In our study, the incidence of MACCE in the TF group was nearly twice as high as in the TR group. This may be explained by the higher prevalence of comorbidities in the TF group, including CKD, dialysis, PAD, and a previous CABG, as well as lower EF, a greater total number of stents, and longer stent lengths and procedural time. These findings suggest that the TF group may have had more severe lesions and included higher-risk patients. As a result, when statistically adjusted for these factors, no significant difference was observed between the two groups. Also, it is possible that the TR approach with RA was performed by operators with more experience in RA, a procedure known to be highly operator-dependent [4,24]. Additionally, changes in RA strategies and improvement of devices may have contributed to the lack of statistical differences in MACCE. Initially used for removing plaque bulk, RA has since transitioned into a technique for modifying plaque [25,26]. Thus, the utilization of 2.0 mm burrs, which require a 7 Fr guiding catheter, has significantly decreased. With the improvement of sheathless guiding catheters and slender sheaths that have smaller external diameters, rotational atherectomy using larger burr sizes can now be safely performed through the radial artery [27]. Thus, the utilization of 2.0 mm burrs, which require a 7 Fr guiding catheter, has significantly decreased. Even in cases where a 7 Fr catheter is required, performing RA with all burr sizes via the radial approach remains feasible [28]. In our study as well, there was no statistically significant difference in the burr sizes used between the TR and TF approaches (1.50 ± 0.20 vs. 1.50 ± 0.20, *p* = 0.367). Given these developments, the heterogeneity of atherosclerotic disease across different vascular beds is an important consideration. Recent studies have emphasized that plaque morphology, vulnerability, and clinical expression vary depending on the anatomical location of the lesion [29]. Such differences support the need for individualized interventional strategies in complex coronary artery disease. RA, as a lesion-specific tool, is particularly well suited to address these diverse pathophysiologic presentations.

In the case of bleeding events during RA, several previous studies comparing the TR and TF approaches have demonstrated that bleeding events were less frequent with the TR approach [21,30]. However, in our study, the total bleeding events did not differ between the two groups. This can be attributed to several reasons. There was a higher proportion of patients on direct oral anticoagulants (DOACs) in the TR group. Additionally, our study was conducted more recently compared to previous studies that demonstrated differences in bleeding events, during which advancements in closure devices have significantly improved hemostasis [31]. Similarly, increased physician experience with hemostasis, driven by the growing use of larger sheaths for procedures such as transcatheter aortic valve implantation, may have contributed. Lastly, our analysis included not only procedure-related bleeding events but also non-procedure-related bleeding events. In addition to bleeding outcomes, periprocedural MI is known to be associated with adverse outcomes and may be considered a potential risk during RA, particularly in complex coronary lesions. However, in our study, the incidence of periprocedural MI was low in both groups. This finding supports the safety of the RA technique even in the setting of complex coronary lesions [32].

This study has several limitations. The main limitation of this study is its retrospective design and the non-randomized selection of the approach sites. While multivariate Cox regression analysis was employed to minimize confounding variables, the possibility of residual confounders influencing the results cannot be fully excluded. Additionally, operator bias based on their prior experience with either the TR or TF approach may have affected the outcomes to some extent [33]. Furthermore, the relatively small sample size reduces the statistical power to detect significant differences. Lastly, the lack of follow-up evaluation, such as coronary angiography, may have led to an underestimation of post-procedure event rates.

## Figures and Tables

**Table 1:** Baseline characteristics.

	TR Approach (*n* = 248)	TF Approach (*n* = 292)	*p*-Value
Age, years	72.5 ± 9.4	70.5 ± 10.6	0.024
Sex			0.069
Male	138 (55.7)	185 (63.4)	
Female	110 (44.3)	107 (36.6)	
BMI	24.6 ± 3.8	24.0 ± 4.0	0.073
Smoking	39 (15.7)	64 (21.9)	0.068
HTN	184 (74.2)	231 (79.1)	0.177
DM	132 (53.2)	173 (59.3))	0.160
Dyslipidemia	110 (44.4)	125 (42.8)	0.718
Family history of CAD	5 (2.0)	6 (2.1)	0.975
CKD	28 (11.3)	68 (23.3)	<0.001
Dialysis	4 (1.6)	45 (15.4)	<0.001
Previous PCI	54 (21.8)	85 (29.1)	0.052
Previous CABG	4 (1.6)	20 (6.9)	0.003
Previous MI	24 (9.7)	42 (14.4))	0.096
CVA	36 (14.5)	39 (13.4)	0.698
PVD	10 (4.0)	29 (9.9)	0.008
Heart failure	22(8.9)	55(18.8)	0.001
Atrial fibrillation	25 (10.1)	24 (8.2)	0.453
Clinical diagnosis			0.558
Stable angina	80 (32.3)	94 (32.2)	
Unstable angina	87 (35.1)	88 (30.1)	
NSTEMI	60 (24.2)	74 (25.3)	
STEMI	6 (2.4)	13 (4.5)	
Silent ischemia	15 (6.1)	22 (7.5)	
Triglyceride	120.4 ± 69.8	119.0 ± 77.7	0.838
Total cholesterol	147.2 ± 35.9	140.4 ± 40.8	0.047
LDL cholesterol	88.6 ± 44.6	81.3 ± 34.1	0.048
HDL cholesterol	46.7 ± 14.7	45.5 ± 14.4	0.350
hsCRP	2.0 ± 4.8	3.9 ± 12.5	0.107
HbA1c	6.8 ± 1.4	6.6 ± 1.4	0.240
Hemoglobin	12.6 ± 2.9	12.1 ± 1.9	0.014
Platelet count	225.3 ± 67.8	214.0 ± 73.3	0.065
Drug			
DOAC	12 (4.8)	4 (1.4)	0.018
DAPT	241 (97.2)	278 (95.2)	0.238
Aspirin	241 (97.2)	288 (98.6)	0.234
P2Y12 inhibitor	244 (98.4)	286 (98.0)	0.760
Statin	231 (93.2)	271(92.8)	0.879

BMI = body mass index; CABG = coronary artery bypass graft; CKD = chronic kidney disease; CVA = cerebrovascular accident; DAPT = dual antiplatelet therapy; DM = diabetes mellitus; DOAC = direct oral anticoagulant; HbA1c = glycated hemoglobin; HDL = high-density lipoprotein; hsCRP = high-sensitivity C-reactive protein; HTN = hypertension; LDL = low-density lipoprotein; MI = myocardial infarction; NSTEMI = non-ST-elevation myocardial infarction; PCI = percutaneous coronary intervention; PVD = peripheral vascular disease; STEMI = ST-elevation myocardial infarction; TF = transfemoral; TR = transradial.

**Table 2:** Baseline angiographic features and procedural specifics.

	TR Approach (*n* = 248)	TF Approach (*n* = 292)	*p*-Value
Lesion classification			0.823
A, n (%)	1 (0.4)	2 (0.7)	
B1, n (%)	20 (8.1)	20 (6.9)	
B2, n (%)	26 (10.5)	26 (8.9)	
C, n (%)	201 (81.1)	244(83.6)	
Vessel diseases			0.815
1VD, n (%)	56 (22.6)	60 (20.6)	
2VD, n (%)	76 (30.7)	95 (32.5)	
3VD, n (%)	116 (46.8)	137 (46.9)	
MVD, n (%)	192 (77.4)	232 (79.5)	0.567
LM disease	31 (12.5)	43 (14.7)	0.454
pre EF	55.5 ± 12.2	50.9 ± 14.0	<0.001
Use of IVUS	120 (48.4)	129 (44.2)	0.328
Mean stent diameter, mm	3.0 ± 0.4	3.0 ± 0.4	0.270
Total number of stent, mm	2.2 ± 1.1	2.5 ± 1.2	0.001
Total stent length, mm	61.8 ± 32.4	70.7 ± 35.2	0.004
Max burr size, mm	1.50 ± 0.20	1.50 ± 0.20	0.367
Procedure time, min	72.4 ± 38.1	85.1 ± 58.7	0.004
Procedure success, n (%)	242 (97.6)	278 (95.2)	0.145

EF = ejection fraction; IVUS = intravascular ultrasound; LM = left main; MVD = multivessel disease; TF = transfemoral; TR = transradial.

**Table 3:** In-hospital events and procedural outcomes.

	TR Approach (*n* = 248)	TF Approach (*n* = 292)	*p*-Value
In-hospital events			
In-hospital MACCE, n (%)	28 (11.3)	32 (11.0)	0.903
In-hospital death, n (%)	5 (2.0)	6 (2.1)	0.975
Urgent CABG/PCI, n (%)	2 (0.8)	7 (2.4)	0.189
Peri-procedural MI, n (%)	21 (8.5)	24 (8.2)	0.917
In-hospital CVA, n (%)	1 (0.4)	1 (0.3)	>0.999
Procedural outcomes			
Coronary dissection, n (%)	20 (8.0)	26 (9.0)	0.680
Temporary pacemaker during procedure, n (%)	2 (0.8)	14 (4.8)	0.007
Coronary perforation, n (%)	4 (1.6)	6 (2.1)	0.760
In-hospital bleeding, n (%)	8 (3.2)	19 (6.5)	0.081

CABG = coronary artery bypass grafting; CVA = cerebrovascular accident; MACCE = major adverse cardiovascular and cerebral event; MI = myocardial infarction; PCI = percutaneous coronary intervention; TF = transfemoral; TR = transradial.

**Table 4:** Clinical outcomes of the TR versus the TF approach.

	TR Approach (*n* = 248)	TF Approach (*n* = 292)	*p*-Value	Univariate HR (95% CI)	*p*-Value	Multivariate HR ** (95% CI)	*p*-Value
MACCE	28 (11.3)	52 (17.8)	0.034	1.643 (1.038–2.601)	0.034	1.520 (0.889–2.600)	0.126
All cause of death	20 (8.1)	26 (8.9)	0.728	1.124 (0.627–2.013)	0.695	1.230 (0.610–2.482)	0.562
Cardiac death	10 (4.0)	20 (6.9)	0.154	1.732 (0.811–3.700)	0.156	1.699 (0.686–4.205)	0.252
MI	7 (2.8)	13 (4.5)	0.318	1.639 (0.654–4.108)	0.292	1.601 (0.536–4.786)	0.400
RR	21 (8.5)	33 (11.3)	0.274	1.399 (0.809–2.417)	0.230	1.140 (0.615–2.114)	0.676
TVR	16 (6.5)	29 (9.9)	0.145	1.622 (0.881–2.986)	0.121	1.212 (0.606–2.424)	0.587
TLR	13 (5.2)	25 (8.6)	0.133	1.706 (0.873–3.334)	0.118	1.245 (0.582–2.663)	0.572
CVA	4 (1.6)	6 (2.1)	0.760	1.301 (0.367–4.610)	0.684	1.479 (0.230–9.512)	0.681
ST	2 (0.8)	5 (1.7)	0.461	2.163 (0.420–11.151)	0.356	2.224 (0.343–14.414)	0.402
Total bleeding	13 (5.2)	19 (6.5)	0.535	1.280 (0.632–2.592)	0.493	1.214 (0.526–2.802)	0.649

** Adjusted by age, sex, CKD, dialysis, previous CABG, PVD, history of heart failure, EF, hemoglobin, total cholesterol, LDL cholesterol. Data are shown as mean ± SD or n (%) CI = confidence interval; CVA = cerebrovascular accident; HR = hazard ratio; MACCE = major adverse cardiovascular and cerebrovascular event; MI = myocardial infarction; RR = repeat revascularization; ST = stent thrombosis; TF = transfemoral; TLR = target lesion revascularization; TR = transradial; TVR = target vessel revascularization.

## Data Availability

Data are available from the corresponding author upon reasonable request.
